# Unraveling diarrheal disease knowledge, understanding, and management practices among climate change vulnerable coastal communities in Ghana

**DOI:** 10.3389/fpubh.2024.1352275

**Published:** 2024-06-14

**Authors:** Yaw Agyeman Boafo, Fidelia N. A. Ohemeng, Jesse Ayivor, Joseph Armah Ayitiah, Dzidzo Yirenya-Tawiah, Adelina Mensah, Cecilia Datsa, Ted Yemoh Annang, Lois Adom

**Affiliations:** ^1^Centre for Climate Change and Sustainability Studies, College of Basic and Applied Sciences, University of Ghana, Legon, Accra, Ghana; ^2^Department of Sociology, College of Humanities, University of Ghana, Legon, Greater Accra, Ghana; ^3^Institute of Environment and Sanitation Studies, College of Basic and Applied Sciences, University of Ghana, Legon, Accra, Ghana

**Keywords:** coastal communities, diseases, households, climate change, diarrhea, Ghana, health

## Abstract

**Introduction:**

Diarrheal disease is a global public health concern, particularly in low-income countries. In Ghana, widespread issues like inadequate sanitation, unsafe drinking water, malnutrition, and poor hygiene practices contribute to the high incidence of diarrhea. Climate change exacerbates these challenges by increasing the frequency and severity of conditions that spread diarrheal diseases. This study explores households’ knowledge, understanding, and management practices for diarrhea in climate change-vulnerable coastal communities.

**Methods:**

The study is set in Ghana’s central (Mumford, Opetekwei) and eastern (Anyako, Anyanui-Atiteti) coastlines. Using a cross-sectional study design, a structured questionnaire was administered to randomly sampled households (*n* = 419) to collect quantitative data. The study collected qualitative data from focus group discussions (*n* = 8), with groups separated into men and women, key informant interviews, and observations of food, water, and sanitation conditions across the studied communities.

**Results and discussion:**

The study found significant variations between the studied communities and socio-demographic variables except for the respondents’ gender. Multivariate regression analyses identified significant associations between socio-demographic variables (especially gender and educational status) and perceptions of diarrhea causes. The most used first management action against diarrhea is ‘over-the-counter drugs’, followed by home-made traditional remedies. Significant differences were observed in the usage of management practices across the studied communities. Trust, affordability, and availability were identified as the main factors influencing households’ use of approved pharmaceutical drugs and traditional herbal remedies for managing behavior, with significant differences being observed across communities. The study recommends a multi-sectoral approach, including improved access to regularly flowing, safe water and sanitation facilities, education on preventing diarrhea, and adequate healthcare services. Community-based interventions such as promoting good hygiene practices at homes and community settings such as schools, lorry parks, funeral grounds, and recreational areas can also effectively reduce the burden of diarrhea.

## 1 Introduction

Diarrhea is one of the climate-sensitive global public health diseases gaining attention in climate change and health research in recent times (1–3). Global estimates report that 1.6 million people across all age groups die annually from diarrheal diseases, responsible for 15% of all under-five deaths (4, 5). As high as 94% of diarrheal disease is attributable to the environment and associated risk factors such as unsafe drinking water, poor socio-economic status, lack of proper sanitation, and poor hygiene (6–10). Other factors also include the type of pathogen, sociocultural practices, behaviors and accessibility, and quality of health systems (11, 12).

Sub-Saharan Africa (SSA) is disproportionately affected by diarrheal diseases, with 86% of reported cases and mortality from cholera (13). Globally, 24% of reported deaths came from the region in 2018 (14), with countries such as the Democratic Republic of Congo, Mozambique, Zambia, Tanzania, Cameroon, Kenya, and Zimbabwe reporting high levels of recurrent cholera occurrences in 2017 and 2018 (14). According to Asamoah et al. (15), diarrhea is responsible for over 14,000 deaths of young children each year in Ghana, making it the third leading cause of death among children under five. The World Health Organization has also reported that the prevalence of diarrheal diseases in Ghana was 6.8% in 2019, with each person experiencing one episode yearly (16). Poor sanitation, contaminated water, malnutrition, and inadequate hygiene practices are some of the reasons for diarrheal diseases in SSA (17–20).

Recent studies have revealed that climate change significantly impacts the prevalence of diarrheal diseases worldwide (2, 21). In Nepal, a study by Dhimal et al. (22) showed that an observed 1° C increase in mean temperature and an increased rainfall of 1 cm resulted in a considerable rise in the incidence of diarrheal illness. This study further estimated that between 2000 and 2010, approximately 800,000 deaths due to diarrhea were attributable to climate change across different regions of Nepal. Hashizume et al. (23) found non-cholera diarrhea cases increased by 5.1% for every 10 mm increase above the threshold of 52 mm of average rainfall over lags 0–8 weeks as well as by 3.9% for every 10 mm decrease below the same threshold of rains. The connection between rainfall-related climate disasters, including flooding, drought, and diarrhea prevalence, has been well-established (24, 25).

In Ghana, flood-exposed communities have been associated with cholera and non-cholera diarrheal disease outbreaks (26–28). As climate change results in more severe and frequent flooding, water source contamination and the spread of waterborne diseases are expected to increase. Abu and Codjoe (6), in their study conducted in two flood-prone and low-income areas, namely James Town and Agbogbloshie within the Accra Metropolitan Area, revealed that households experience regular cholera outbreaks and a high prevalence of non-cholera diarrhea and other illnesses. These low-income communities also have a large percentage of children under 5 years old (29) who may have weakened immune systems and be particularly vulnerable to environmental challenges.

Several studies have in recent years pointed out the significant impacts of climate change on coastal communities (30, 31). The increasing vulnerability of coastal communities to climate change worsens the risks associated with diarrheal diseases (32). Coastal communities in Ghana are vulnerable to the effects of climate change (33–35) and at risk of exacerbated poor health and well-being because of climate-induced effects such as flooding from rain, sea level rise and storm surges, coastal erosion, poor environmental sanitation, limited access to safe drinking water, and inadequate hygiene practices (36–38). In addition, rising sea levels and more intense storms are expected to contribute to contaminating coastal waters with pollutants and sewage (32, 39), thus making diarrheal diseases one of the biggest health threats and concerns facing households.

The management of diarrheal diseases in SSA and, for that matter, coastal communities is still a significant public health challenge (40, 41), despite the availability of effective interventions such as oral rehydration therapy, zinc supplementation, and improved hygiene and sanitation practices. Limited access to trained healthcare providers, overuse of antibiotics, and inadequate hygiene and sanitation practices contribute to the burden of diarrheal diseases, particularly among vulnerable populations in coastal landscapes (42, 43). Our survey of the existing literature shows no available data on how climate change-vulnerable coastal communities manage diarrheal diseases in Ghana. The absence of available data on the management of diarrheal diseases in climate change-vulnerable coastal communities of Ghana poses a significant threat to the design and implementation of effective strategies to combat diarrheal diseases and improve the overall health and resilience of coastal households and communities in the context of climate change.

The primary objective of this study was to examine the experiences of diarrheal diseases and the corresponding management practices employed within climate change-vulnerable coastal communities in Ghana. Developing a full understanding of the factors that shape these management behaviors can play a critical role in enhancing our knowledge of diarrheal disease prevention and management, especially in the face of accelerating climate change impacts in coastal communities in Ghana. The insights gained from this study hold great value for healthcare providers, enabling them to diagnose and treat diarrheal diseases more effectively. In addition, policymakers can utilize the findings to develop targeted interventions and policies to improve these communities’ prevention and management of diarrheal diseases.

## 2 Materials and methods

### 2.1 Description of study area and sites

**Figure 1:**
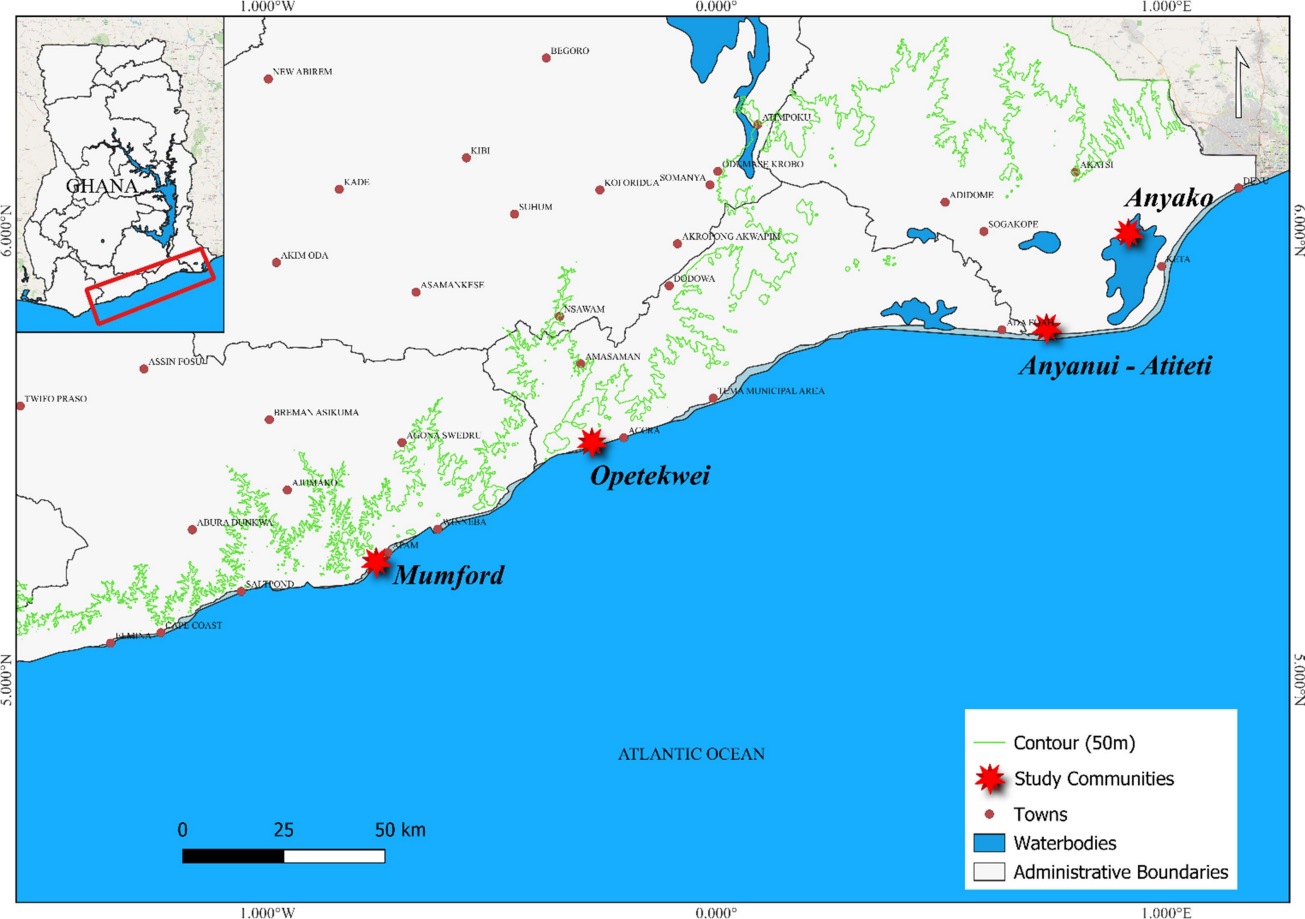
Geographic overview of Ghana highlighting the study districts and communities.

This study is part of a five-year collaborative and interdisciplinary project called *‘Coastal Communities Resilience to Climate and Diarrhea* (C2R-CD; FY2020-2025)’. The C2R-CD project aims to build resilience to climate change and improve diarrheal management in coastal communities. Coastal Ghana, spanning from the border of Togo to Cote D’Ivoire, covers about 560 kilometers along the Gulf of Guinea (44) (Figure 1). Ghana’s coastline is known for its diverse marine biodiversity, coastal wetlands, cultural heritage, and various communities with distinct ethnic groups, languages, and traditions. The major ethnic groups include the Ga-Adangbe, Ewe, Fantes, Nzema, Ahanta, and Anlo.

The socio-economic activities of the coastal communities revolve around fishing, small-scale trading, farming (subsistence), craft production, sand mining, and tourism. Fishing is the primary economic activity, with artisanal fishing using small boats and nets being the most common form, while women dominate the processing and marketing sectors. Farming is also essential, with farmers producing crops mainly for household consumption and selling little in local markets. Tourism provides opportunities for hospitality services, coastal resorts, heritage sites like Cape Coast and Elmina castles, ecotourism, and cultural experiences dotted along Ghana’s coastline.

In recent years, Ghana’s coastal socio-ecological system has become fragile due to the impacts of various human-induced activities, including overfishing and illegal fishing practices (45), illegal sand mining (46, 47), oil exploration, pollution (48), and infrastructure construction to support tourism services (49). The highlighted activities create social, ecological, economic, and health challenges, significantly impacting the well-being of households and communities. In addition to these activities, coastal Ghana is highly vulnerable to the impacts of climate change with sea-level rise, increased frequency and intensity of storm, erosion, and flooding exacerbating existing challenges and resulting in displacement, loss of livelihoods, and rise in environmental health issues like malaria, cholera, and childhood diseases (44). Across many coastal communities in Ghana, poor environmental sanitation management, resulting from inadequate water, waste, and toilet facilities, has also increased the risk of disease transmission (35, 50).

This present study’s sampling area was the entire Ghana’s coastline. To settle on case study communities, the project team undertook a series of reconnaissance surveys to sampled locations in the Volta, Greater Accra, and Central regions of Ghana. During the reconnaissance surveys, the study team explored the following criteria in selecting case study sites: reported incidence of flooding, wave dynamics, presence or absence of health facilities, hygiene and sanitation situation, diarrhea endemism, and population density. Based on these criteria, four main communities were selected. The communities are Anyako and Anyanui-Atiteti in the Volta, Opetekwei in the Greater Accra, and Mumford in the Central regions of Ghana (Figure 1). Principally, these four communities were selected based on (i) previous incidences of diarrhea, (ii) exposure and sensitivity to climate-induced hazards, (iii) overcrowding, (iv) limited access to safe and reliable drinking water, and (v) geographic spread. The social-ecological characteristics of the study sites are provided in Table 1. These communities from our survey are known for their different levels of vulnerability to the impacts of climate change, including erosion, sea level rise, and flooding. These impacts are known to be key drivers of increased water-borne diseases, including diarrhea.

**Table 1:** Socio-ecological profile and key characteristics of the study communities.

Key criteria for selection	Study communities			
	Anyako	Anyanui-Atiteti	Mumford	Opetekwei
Geographic location	Lat 5.9941104°N Lon −0.915794°W	Lat. 5.783375° N Lon −0.733375°W	Lat 5.262484° N Lon −0.758033°W	Lat 5.528831° N Lon −0.277500°W
Region	Volta	Volta	Central	Greater Accra
Municipal/District	Keta Municipal	Anloga District	Gomoa West Municipal	Ablekuma West Municipal
District population*	78,862	94,858	129,512	159,208
Estimated Community Population (2021)	11,079	6,532	17, 561	8,000
Main ethnic groups residing.	Ewe, Mina	Ewe, Mina	Fante, Ashanti, Akuapem, Ewe, Ga-Adangme	Ga-Adangme, Akan, Ewe, Hausa, Dagomba
Main economic activity	Fishing	Fishing	Subsistence farming, Fishing,	Trade, Artisanal activities
Alternate livelihood activity	Subsistence farming, small-scale trading	Subsistence farming (shallot, pepper), Mangrove farming	Trading and market activities, small-scale crafts production	Fishing, Trading,
Average monthly rainfall in the rainy season (mm)	150–200	150–200	150–200	40–120
Temperature	27°C- 34°C	27°C- 34°C	28°C- 35°C	25°C-32°C
Main climate-induced hazards	Flooding, Temperature increases	Flooding, Erosion, Sea level rise, storm surges	Flooding, Erosion, Storm surges,	Flooding, Erosion, and Temperature increases

Source: Field Survey, 2021; Literature review *Ghana Statistical Service, 2022.

### 2.2 Data collection and analyses

The research team used multiple techniques to acquire quantitative and qualitative data from primary and secondary sources to understand communities’ and households’ diarrheal disease experiences and management strategies. The team gathered relevant secondary data from published and unpublished literature from various sources, including libraries, hospital and health center records, municipal and district assembly databases, and the internet.

For the collection of primary data, the study team used household (questionnaire) surveys, key informant interviews, and focus group discussions. A preliminary step in this process was conducting a pilot study in May 2021, specifically in the Opetekwei community, to test the clarity and comprehensibility of the questionnaire and interview guide among potential respondents. This pre-testing involved 15 households to refine the questionnaire’s effectiveness in capturing relevant data. Prior to the commencement of the pilot survey a training program for enumerators was implemented to ensure the accuracy and reliability of the data. This training was designed to equip the enumerators with the necessary skills and knowledge to conduct the interviews and discussions consistently across different households and communities.

The main survey for primary data was conducted between July and August 2021.The standardized household questionnaire, which included both closed and open-ended questions, comprised a range of topics. These topics covered the essential socio-demographic background of households, their sources and access to water and sanitation, knowledge about the causes of diarrhea, experiences of household members with diarrheal diseases, management practices, and the use of both approved and unapproved drugs. We determined the sample size using Cochran’s formula, aiming for a 95% confidence level with a 5% margin of error. This calculation was based on a conservative estimate of a 50% prevalence rate. It suggested a minimum sample size of 385 households across the communities. To account for non-responses and data attrition, we added an 8% buffer. This resulted in an adjusted total sample size divided among Anyako (*n* = 93), Anyanui-Atiteti (*n* = 29), Opetekwei (*n* = 30), and Mumford (*n* = 267), reflecting their proportional population sizes. The proportion of households sampled for the surveys in each community was carefully calculated to ensure a representative and statistically significant cross-section of the population. In Anyako, the proportion was 0.84%; in Anyanui-Atiteti, it was 0.44%; in Mumford, it was 1.52%; and in Opetekwei, it was 0.38%. These proportions were determined with the aim of balancing scientific rigor with logistical feasibility, considering the constraints of the study’s resources and objectives.

Our sampling strategy combined stratified random sampling with systematic and cluster sampling techniques. The communities served as strata, ensuring representation across diverse geographic and demographic lines. Within each community, households were selected systematically, with cluster sampling employed in denser areas to ensure adequate representation of every segment of the population (51). We focused on households with present and consenting adults, excluding those without available consenting members to maintain ethical standards. The interviews were conducted at the respondents’ homes or preferred locations at convenient times and often lasted between 50 min and 1 h.

In this study, a total of eight focus group discussions were conducted across the four communities. Each community hosted two meetings, one for women and another for men (Table 2). Participants for the FGDs were recruited through a purposive sampling method. Eligibility criteria for participants included being a resident of the community for at least 1 year and having direct experience with managing diarrhea, either personally or within their household. All participants were briefed about the study’s aims, their rights as voluntary participants, and the guarantee of confidentiality through informed consent forms. Prior to the sessions, participants were provided with informed consent forms detailing the study’s aims, their rights as voluntary participants, including the right to withdraw at any time without consequence, and the measures taken to ensure confidentiality and anonymity. The discussions were guided by a structured discussion guide developed by the study team. We explored open-ended queries on diarrhea prevalence, causes, and management practices, touching upon access to clean water, sanitation amenities, home remedy methods, and hygiene routines. The facilitator followed this guide, prompting participants to express their views and share personal experiences regarding diarrhea management. Examples of questions from the guide include: “What are the most common practices in your community for managing diarrhea.” The facilitator ensured that discussions were inclusive, allowing each participant the opportunity to contribute, and used probing questions to delve deeper into specific topics as they emerged. The sessions lasted for approximately 1–2 h.

**Table 2:** Distribution of focus group discussants by gender in each community.

Community	No of focus group discussion (FGD) participants		Total
	Men FGD	Women FGD	
Anyako	8	8	16
Anyanui-Atiteti	7	9	16
Mumford	8	10	18
Opetekwei	9	8	17

Source: Field Survey, 2021.

The statistical analysis for this study was performed in IBM SPSS Statistics (version 23) and Python. The collected data was entered into the software and cleaned to ensure accuracy, completeness, and consistency. Descriptive statistics were used to calculate frequency distributions and percentages for categorical variables, providing a clear understanding of the socio-demographic profiles, health practices, and infrastructure use among the households surveyed. Cross-tabulations provided insights into how these variables were distributed across different communities. To assess the statistical significance of observed differences and associations, chi-square tests were used, especially for categorical variables such as educational status, religious affiliation, and primary sources of drinking water across various communities. The significance of these associations was determined by calculating *p*-values, with thresholds set at *p* ≤ 0.05, *p* ≤ 0.01, and *p* ≤ 0.001 to indicate different levels of significance.

To examine the influence of socio-demographic variables on the perceived causes of diarrhea, we conducted a multivariate logistic regression analysis, allowing for the simultaneous evaluation of multiple predictor variables to determine their independent effects on the binary outcome of reporting diarrhea. The dependent variable was the self-reported incidence of diarrhea, while key independent variables included perceptions about specific causes of diarrhea (drinking from the river, eating unwashed fruits, eating uncooked food, food not covered, food not stored well, and flooding effects) and socio-demographic factors (religious affiliation as Christian, gender of household head, and education level with no formal education). Each perception-related variable was coded into agree, disagree, and no idea categories. Three separate logistic regression models were performed to explore interactions between perceptions and socio-demographic factors, adjusted for potential confounders and including interaction terms where necessary. Model 1 examined the impact of perceptions among Christians, Model 2 analyzed the influence based on the sex of the household head, and Model 3 focused on households with no formal education. Odds Ratios (OR) and 95% Confidence Intervals (CI) were calculated to quantify the effects, with *p*-values determining statistical significance. This multivariate approach provided a comprehensive understanding of how socio-demographic variables and health perceptions interact to influence diarrhea incidence, guiding targeted interventions in climate change vulnerable communities.

The qualitative data collected through focus group discussions and key informant interviews were analyzed using thematic analysis. The final themes and patterns were presented in a narrative format and integrated with the quantitative data to provide a comprehensive understanding of diarrhea management practices in coastal communities of Ghana.

### 2.3 Ethical consideration

This study adhered to ethical principles and obtained clearance from the Ethics Committee for the College of Basic and Applied Science (ECBAS 044/19–20) of the University of Ghana and the Ghana Ministry of Health (GHS-ERC-011/11/20) before conducting the household survey and focus group discussions. All eligible participants were fully informed of the purpose of the study, the associated risks and benefits and provided with written information and a consent form. Non-English-speaking participants were given a detailed oral explanation of the research and asked for verbal consent, accompanied by a thumb impression on the consent form in the presence of community members of their choice. Participants were also informed about data anonymization and confidentiality during the informed consent procedure. Before starting the questionnaire survey, all participants gave verbal and written informed consent and were explicitly informed of their right to refuse participation or withdraw at any point without negative consequences. The study followed ethical principles of beneficence, non-malevolence, justice, and equity in collecting data from eligible and consenting participants.

## 3 Results

### 3.1 Socio-demographic characteristics of respondents

The questionnaire survey conducted in the study sites sampled 430 households, out of which 419 completed the survey, resulting in a high response rate of 97%. The relevant socio-demographic characteristics of the respondents were analyzed (Table 3). The results showed that most households (62.5%) were headed by males, while females headed the remaining 37.5%. No statistically significant difference was observed in the distribution of male and female households across the studied communities. Regarding educational status, most households had some formal education (76.6%), with 24.3% having no formal education, 25.9% completing primary education, and 32.7% completing junior high school. Only a small percentage of households (2.3%) had completed tertiary education. The educational status of households shows a significant difference among the communities studied (*p* ≤ 0.001). Primary and middle school education was the highest level achieved by most respondents in Anyako (36.6%) and Anyanui-Atiteti (55%). In Opetekwei, SHS/Tech/Voc was the highest level of education attained by the majority of respondents (40%), followed by JHS/Middle School education (33%). In the Mumford community, the majority of respondents had no formal education (35.6%), followed by those with JHS/Middle School education (28.8%; *p* < 0.001).

**Table 3:** Socio-demographic characteristics of surveyed households in the studied communities.

Variable	Total (*N* = 419) n (%)	Anyako (*N* = 93) n (%)	Anyanui- Atiteti (*N* = 29) n (%)	Opetekwei (*N* = 30) n (%)	Mumford (*N* = 267) n (%)	*p*-value
Sex of households						
*Male*	262 (62.5)	69 (74.2)	15(51.7)	17(56.7)	161(60.2)	0.1026^NS^
*Female*	157 (37.5)	24(25.8)	14(48.3)	13(43.3)	106(39.7)	
Educational status						
*No formal*	102(24.3)	5(5.4)	1(3.4)	1(3.3)	95(35.6)	0.0007***
*Primary*	106 (25.9)	30(32.3)	7(24.1)	5(16.7)	64(24.0)	
*JHS/Middle School*	137(32.7)	34(36.6)	16(55.0)	10(33)p	77(28.8)	
*SHS/Tech/Voc*	64 (15.3)	19(20.4)	5(17.2)	12(40)	28(10.5)	
*Tertiary*	10(2.3)	5(5.4)	0(0)	2(6.7)	3(1.1)	
Religious affiliation						
*Christian*	325(80)	51(54.8)	19(65.5)	30(100)	225(84.2)	0.000***
*Islam*	27(6.0)	0(0)	0(0)	0(0)	25(9.4)	
*Traditional*	69(14)	42(45.0)	10(34.4)	0(0)	17(6.4)	
Main source of drinking water^§^						
*Surface water*	0(0)	0(0)	0(0)	0(0)	0(0)	0.001***
*Boreholes*	11(2.6)	6(6.5)	1(3.4)	0(0)	4(1.5)	
*Pipe-borne water*	289(68)	25(26.9)	22(75.8)	3(10.0)	239(89.5)	
*Rainwater*	14(3.34)	4(4.3)	0(0)	0(0)	10(3.7)	
*Bottled water*	6(1.4)	2(1.1)	1(3.4)	1(3.3)	2(0.7)	
*Sachet water*	316(75.4)	86(92.4)	23(79.3)	30(100)	177(43.8)	
*Tanker supply*	67(15.9)	14(15.0)	5(17.2)	15(50)	33(12.3)	
Main source of water for cooking and handwash^§^						
*Surface water*	16(3.8)	12(13.0)	6(20.6)	0(0)	0(0)	0.001***
*Boreholes*	28(6.7)	23(24.7)	0(0)	3(10.0)	1(0.4)	
*Pipe-borne water*	342(81.6)	34(36.5)	15(79.3)	26(86.7)	259(97.0)	
*Rainwater*	2(0.5)	1(1.0)	0(0)	0(0)	1(0.4)	
*Bottled water*	0(0)	0(0)	0(0)	0(0)	0(0)	
*Sachet water*	8(1.8)	1(1.0)	5(17.2)	18(3.3)	6(2.2)	
*Tanker supply*	68 (16,2)	14(36.5)	0(0)	31 (36.6)	23(8.6)	
Solid waste disposal methods^§^						0.001***
*Dustbin/Dumpster*	116 (22.4)	34 (27.4)	3 (8.1)	30(59.1)	49 (16.1)	
*Refuse Dump (Open spaces)*	328 (63.4)	71 (57.4)	25 (67.6)	12 (23.5)	220 (72.1)	
*Open burning*	73 (14.1)	19 (15.3)	9 (24.3)	9 (17.6)	36 (12.0)	
Types of Toilets used by Household						0.001***
*Flush to a sewer system*	2 (0.4)	0 (0.0)	0 (0.0)	0 (0.0)	2 (0.6)	
*Flush to septic tank*	31 (6.7)	7 (7.5)	2 (6.5)	12 (40.0)	10 (3.2)	
*Flush to a pit latrine*	15 (3.2)	5 (5.4)	2 (6.5)	1 (3.3)	7 (2.3)	
*Kumasi Ventilated Improved Pit Latrine (KVIP)*	27 (5.8)	6 (6.5)	4 (12.9)	3(10.0)	14 (4.5)	
*Pit latrine with slab*	6 (1.3)	2 (2.2)	1 (3.2)	1(3.3)	2 (0.6)	
*Pit latrine without a slab*	14 (3.0)	3 (3.2)	0 (0.0)	9 (30.0)	2 (0.6)	
*Open defecation*	129 (27.8)	2(2.2)	2 (6.5)	0 (0.0)	125 (40.3)	
*Public toilet*	240 (51.7)	68 (73.1)	20 (64.5)	4 (13.3)	148 (47.7)	

§Respondents could select multiple options that apply to their households hence the total count of responses exceeds the number of respondents. NS, Not significant; *p* < 0.05: *(statistically significant, 95% confidence); *p* < 0.01: **(highly statistically significant, 99% confidence); *p* < 0.001: ***(very highly statistically significant, 99.9% confidence). Source: Field Survey, 2022.

Regarding religious affiliation, 80% of participants identified as Christians, 14% as Traditionalists, and 6% as Muslims. The religious affiliation of households varied significantly across the communities (*p* ≤ 0.001). The proportion who asserts to be part of Islam was low in all communities, with only 9.4% identified in the study. The highest proportion of traditionalists was recorded in the Anyako community, with 45%, while the studied communities had proportions ranging from 0 to 34.4%.

With regards to households and communities’ primary source of drinking water, the survey results showed that sachet water (“pure water” as is popularly called) is the most used source of drinking water by the majority of households in all the communities studied (Mean = 75.4%; Opetekwei = 100%; Anyako = 92.4%; Anyanui-Atiteti = 79.3%) except for Mumford where the majority (89.5%) reported pipe-borne water. Only a small proportion of households—ranging from 0.7 to 3.4% across different communities—use bottled water. Tanker water is also a key source of water for drinking, according to surveyed households, especially for respondents in Opetekwei (50%). The primary source of drinking water shows a significant difference among communities (*p* ≤ 0.001).

The question on household’s main source of water for cooking and handwashing revealed that pipe-borne water is the most used (81.6% overall). Approximately 16 % (16.2%) of households reported having a tanker supply water for cooking and handwashing. Mumford has the highest percentage of households relying on pipe-borne water (97.0%). In contrast, Opetekwei had a higher percentage (50.0%) of households relying on tanker supply, suggesting water scarcity or infrastructure challenges in that community. Anyako has a higher percentage of households relying on boreholes (24.7%) for cooking and handwashing. The results indicate a significant difference among the studied communities (*p* ≤ 0.001).

According to the survey results, the most common waste management method is the refuse dump, used by 63.4% of households, followed by dustbin or dumpster (22.4%) and open burning (14.1%). How households manage solid waste showed a significant difference among communities in coastal Ghana. For example, in Opetekwei, the majority of households (59.1%) used a dustbin, while in Anyanui-Atiteti, the majority (67.6%) used a refuse dump. Open burning was most common in Anyanui-Atiteti (24.3%), while it was less common in Opetekwei (17.6%; *p* ≤ 0.001).

Regarding the type of toilet facility households use, the results indicate that public toilets are the most used system, with a usage rate of 51.7% overall. Open defecation is still practiced by a significant proportion of households at an overall usage rate of 27.8%, followed by the flush-to-septic tank (6.7%), KVIP (5.8%), and flush-to-pit latrine system (3.2%). However, the usage rates of these facilities vary significantly across communities (*p* ≤ 0.001). Mumford has the highest percentage of households using flush toilets connected to septic tanks (40%), while Anyako has the highest percentage of households practicing open defecation (40.3%). Anyanui-Atiteti has a higher percentage of households using public toilets (64.5%).

### 3.2 Influence of key socio-demographic variables on causes of diarrhea

The multivariate logistic regression analysis reveals significant associations between socio-demographic variables and the perceived causes of diarrhea in the studied coastal communities (Table 4). In the first model examining perceptions among Christians, individuals who disagreed with drinking from the river had significantly lower odds of experiencing diarrhea (OR = 0.34, 95% CI: 0.18 to 0.63, *p* = 0.037). Disagreement about covering food was associated with higher odds of diarrhea (OR = 3.41, 95% CI: 1.43 to 8.12, *p* = 0.005). Similarly, those who disagreed that food was stored well had lower odds of diarrhea (OR = 0.23, 95% CI: 0.06 to 0.88, *p* = 0.031). Additionally, disagreement with the effects of flooding significantly increased the odds of diarrhea (OR = 4.14, 95% CI: 1.01 to 17.10, *p* = 0.048). Individuals who had no idea about eating unwashed fruits had markedly higher odds of diarrhea (OR = 7.07, 95% CI: 1.50 to 33.23, *p* = 0.003), while those with no idea about eating uncooked food had lower odds (OR = 0.31, 95% CI: 0.14 to 0.70, *p* = 0.009).

**Table 4:** Multivariate logistic regression results on causes of diarrhea by socio-demographic variables.

Variable (Group)	OR	95% CI	*p*-value
*Model 1: Perceptions among Christians*			
Drinking from river (Agree)	1.00	-	-
Drinking from river (Disagree)	0.34	0.18 to 0.63	0.037*
Food not covered (Agree)	1.00	-	-
Food not covered (Disagree)	3.41	1.43 to 8.12	0.005**
Food not stored well (Agree)	1.00	-	-
Food not stored well (Disagree)	0.23	0.06 to 0.88	0.031*
Flooding effects (Agree)	1.00	-	-
Flooding effects (Disagree)	4.14	1.01 to 17.10	0.048*
Eating unwashed fruits (No)	1.00	-	-
Eating unwashed fruits (No Idea)	7.07	1.50 to 33.23	0.003**
Eating uncooked food (No)	1.00	-	-
Eating uncooked food (No Idea)	0.31	0.14 to 0.70	0.009**
*Model 2: Influence Based on the Sex of the Household Head*			
Drinking from river (Male, Agree)	1.00	-	-
Drinking from river (Male, Disagree)	0.61	0.38 to 0.97	0.019*
Drinking from river (Female, Agree)	1.00	-	-
Drinking from river (Female, Disagree)	0.78	0.32 to 1.93	0.591
Food not covered (Male, Agree)	1.00	-	-
Food not covered (Male, Disagree)	1.95	1.02 to 3.73	0.041*
Food not covered (Female, Agree)	1.00	-	-
Food not covered (Female, Disagree)	3.87	1.56 to 9.62	0.004**
Food not stored well (Male, Agree)	1.00	-	-
Food not stored well (Male, Disagree)	0.67	0.23 to 1.97	0.467
Food not stored well (Female, Agree)	1.00	-	-
Food not stored well (Female, Disagree)	0.18	0.04 to 0.76	0.021*
Flooding effects (Male, Agree)	1.00	-	-
Flooding effects (Male, Disagree)	3.18	1.05 to 9.63	0.039*
Flooding effects (Female, Agree)	1.00	-	-
Flooding effects (Female, Disagree)	5.12	1.89 to 13.89	0.002**
Eating unwashed fruits (Male, No)	1.00	-	-
Eating unwashed fruits (Male, Disagree)	4.22	1.55 to 11.48	0.006**
Eating unwashed fruits (Female, No)	1.00	-	-
Eating unwashed fruits (Female, No Idea)	6.73	2.08 to 21.73	0.001**
Eating uncooked food (Male, No)	1.00	-	-
Eating uncooked food (Male, Disagree)	0.48	0.25 to 0.92	0.022*
Eating uncooked food (Female, No)	1.00	-	-
Eating uncooked food (Female, No Idea)	0.23	0.06 to 0.86	0.030*
*Model 3: Households with No Formal Education*			
Drinking from river (No Formal Education, Agree)	1.00	-	-
Drinking from river (No Formal Education, Disagree)	2.54	1.12 to 5.76	0.026*
Food not covered (No Formal Education, Agree)	1.00	-	-
Food not covered (No Formal Education, Disagree)	2.00	1.10 to 3.64	0.021*
Food not stored well (No Formal Education, Agree)	1.00	-	-
Food not stored well (No Formal Education, Disagree)	0.31	0.11 to 0.90	0.044*
Flooding effects (No Formal Education, Agree)	1.00	-	-
Flooding effects (No Formal Education, Disagree)	2.38	1.12 to 5.06	0.023*
Eating unwashed fruits (No Formal Education, No)	1.00	-	-
Eating unwashed fruits (No Formal Education, No Idea)	7.07	1.50 to 33.23	0.003**
Eating uncooked food (No Formal Education, No)	1.00	-	-
Eating uncooked food (No Formal Education, No Idea)	0.31	0.14 to 0.70	0.009**

*p* < 0.05: *(statistically significant, 95% confidence); *p* < 0.01: **(highly statistically significant, 99% confidence).

In the second model focusing on the sex of the household head, males who disagreed with drinking from the river had significantly lower odds of experiencing diarrhea (OR = 0.61, 95% CI: 0.38 to 0.97, *p* = 0.019). Disagreement about covering food increased the odds of diarrhea for both males (OR = 1.95, 95% CI: 1.02 to 3.73, *p* = 0.041) and females (OR = 3.87, 95% CI: 1.56 to 9.62, *p* = 0.004). Females who disagreed with proper food storage had significantly lower odds of diarrhea (OR = 0.18, 95% CI: 0.04 to 0.76, *p* = 0.021). Disagreement about the effects of flooding significantly increased the odds of diarrhea for both males (OR = 3.18, 95% CI: 1.05 to 9.63, *p* = 0.039) and females (OR = 5.12, 95% CI: 1.89 to 13.89, *p* = 0.002). Males who disagreed about eating unwashed fruits had higher odds of diarrhea (OR = 4.22, 95% CI: 1.55 to 11.48, *p* = 0.006), and females who had no idea about eating unwashed fruits also had higher odds (OR = 6.73, 95% CI: 2.08 to 21.73, *p* = 0.001). Finally, males who disagreed about eating uncooked food had lower odds of diarrhea (OR = 0.48, 95% CI: 0.25 to 0.92, *p* = 0.022), and females with no idea about eating uncooked food also had lower odds (OR = 0.23, 95% CI: 0.06 to 0.86, *p* = 0.030).

In the third model examining households with no formal education, those who disagreed with drinking from the river had higher odds of experiencing diarrhea (OR = 2.54, 95% CI: 1.12 to 5.76, *p* = 0.026). Disagreement about covering food also increased the odds of diarrhea (OR = 2.00, 95% CI: 1.10 to 3.64, *p* = 0.021), while disagreement about proper food storage decreased the odds (OR = 0.31, 95% CI: 0.11 to 0.90, *p* = 0.044). Disagreement about the effects of flooding was associated with higher odds of diarrhea (OR = 2.38, 95% CI: 1.12 to 5.06, *p* = 0.023). Additionally, having no idea about eating unwashed fruits significantly increased the odds of diarrhea (OR = 7.07, 95% CI: 1.50 to 33.23, *p* = 0.003), while having no idea about eating uncooked food decreased the odds (OR = 0.31, 95% CI: 0.14 to 0.70, *p* = 0.009).

**Figure 2:**
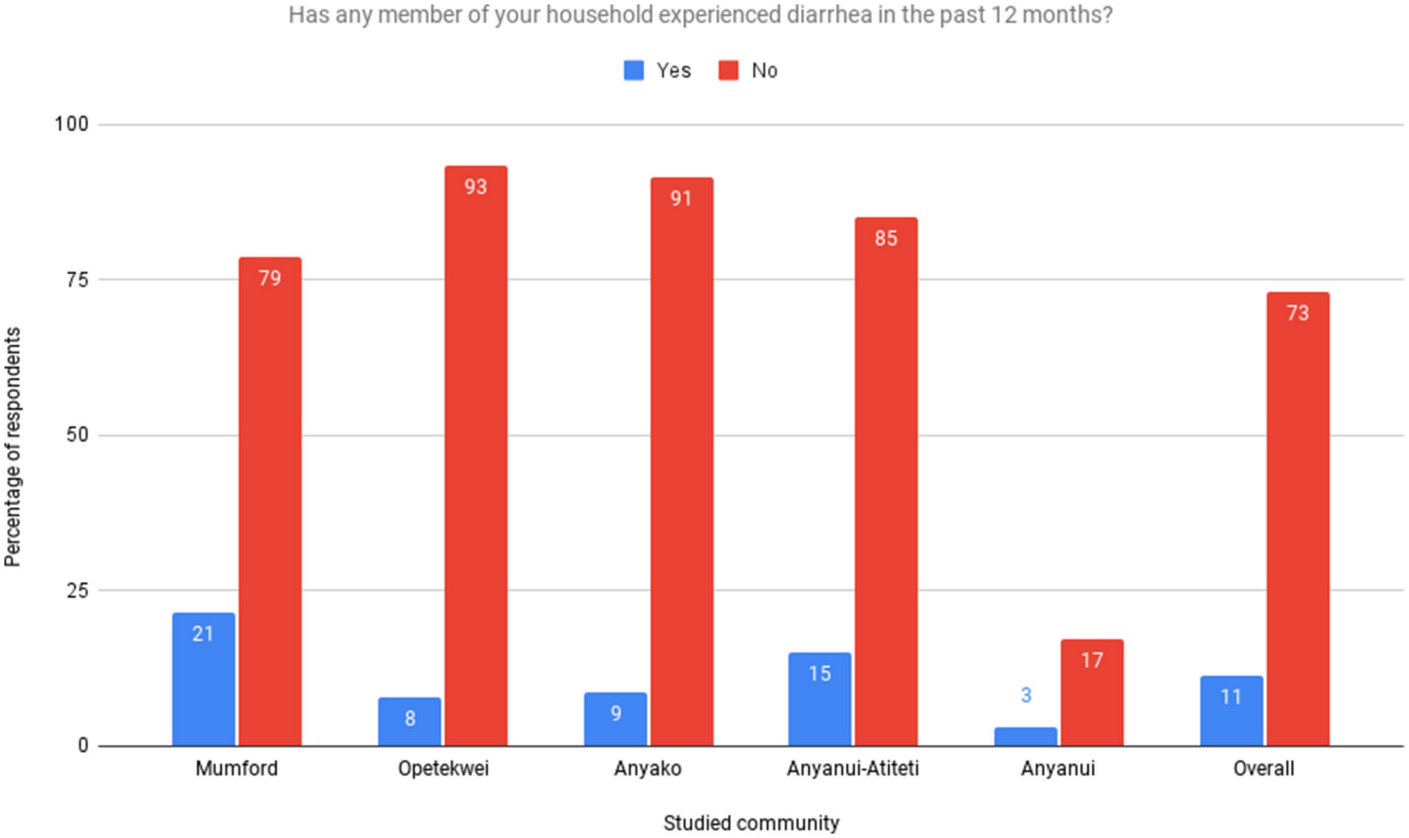
Has any member of your household experienced diarrhea in the past 12 months. Source: Field Survey, 2021.

In the analysis of households’ experiences with diarrheal diseases over the past 12 months (Figure 2), it was found that a minimal percentage of households reported experiencing diarrhea. However, there were noticeable variations in the incidence of diarrhea across the different communities studied. Mumford had the highest percentage of households (21.3%) that have experienced diarrhea, followed by Anyanui-Atiteti (15%), Anyako (8.6%), and Opetekwei (7.7%). On the other hand, the overall percentage of households that have experienced diarrhea in the past 12 months is 13.15%, a moderate prevalence level.

### 3.3 Diarrhea management practices in the studied communities

#### 3.3.1 First management action for diarrheal diseases

**Figure 3:**
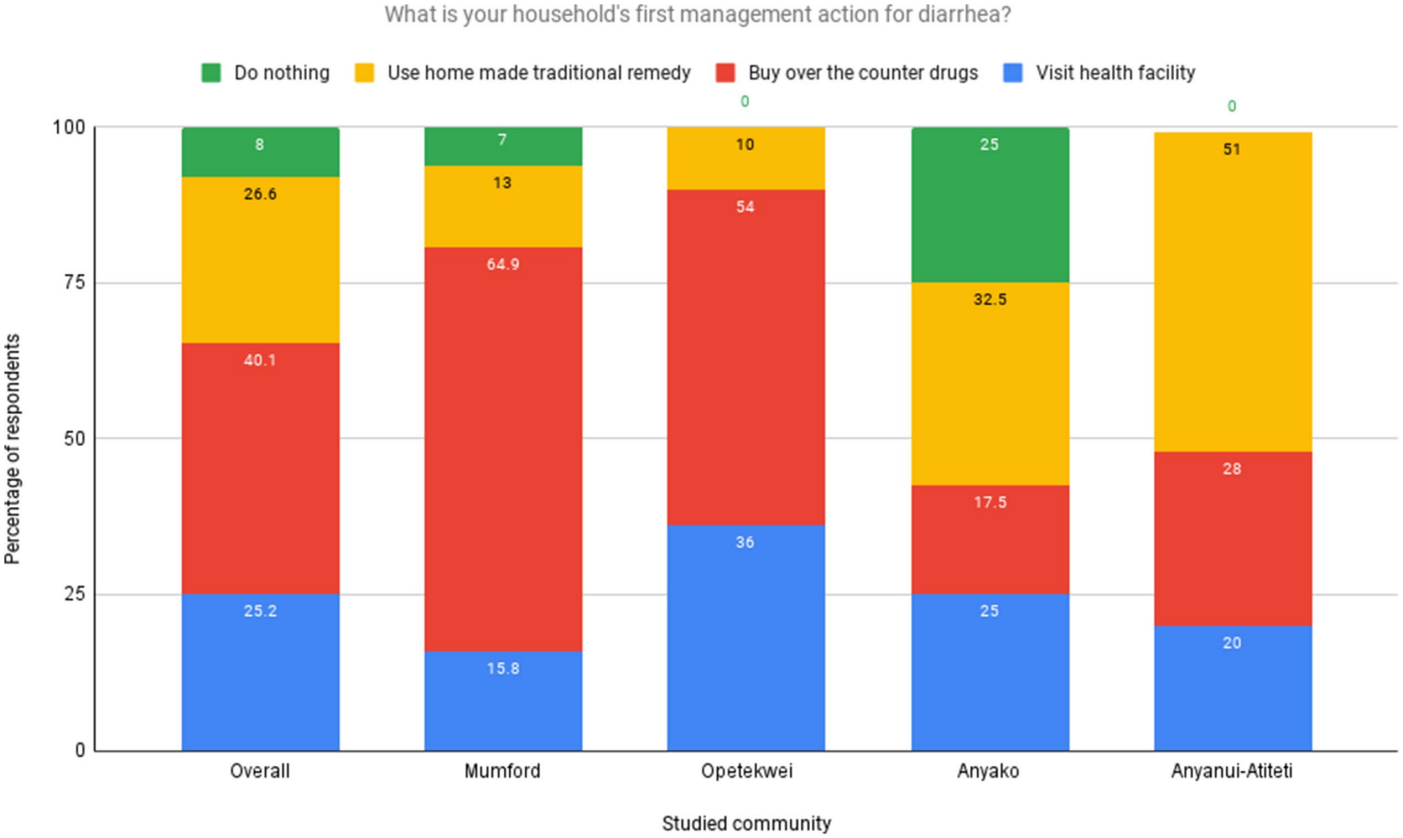
First management action taken by households for diarrheal diseases. Source: Field Survey, 2021.

From the results (Figure 3), ‘buy over-the-counter drugs’ is the most commonly (40.1%) used first management action by households across the studied coastal communities. The ‘use of traditional home-made remedy’ is the second most reported first management option (26.6%) of households, whereas ‘visit to health facility’ is the third most used action by households interviewed (25.2%).

**Figure 4:**
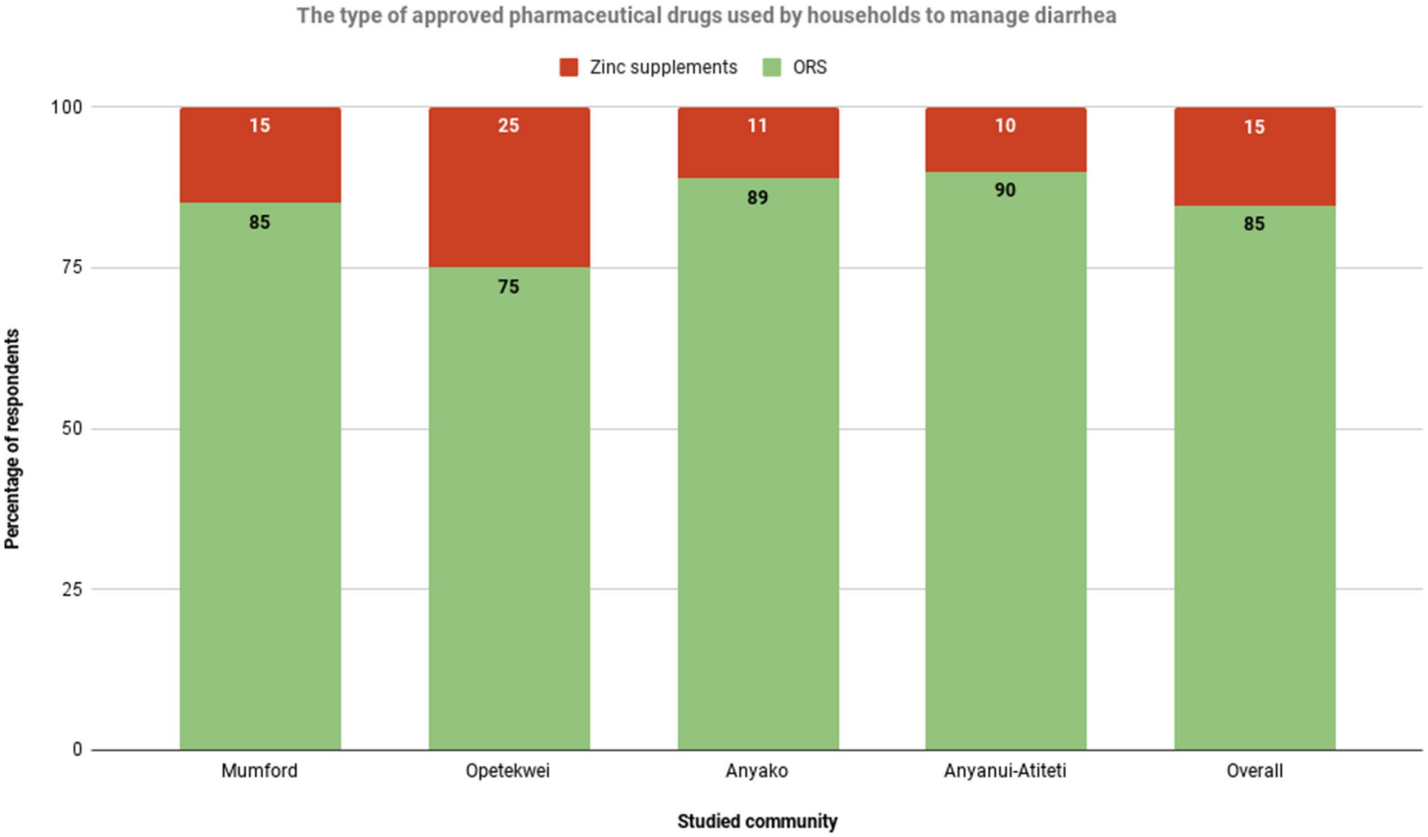
Households’ usage rate of approved pharmaceutical diarrhea drugs. Source: Field Survey, 2021.

When stratified by communities, most households from Mumford (64.9%) and Opetekwei (54%) rely on over-the-counter drugs as a first management action. Most in Anyanui-Atiteti (51%) and Anyako (32.5%) use traditional homemade remedies as the first management option for managing diarrhea. Visiting health facilities is widely used by households, with significant reports across all the studied sites, with a substantial proportion of households in Opetekwei (36%) reporting it (Figure 4). The findings from the focus group discussions conducted across the study communities provided valuable insights that aligned with the responses obtained from the household survey.

In the Mumford community, several discussants emphasized the importance of over-the-counter drugs and home-made traditional remedies for managing diarrhea. In the words of a 43-year-old discussant.

“*For me, the first thing I do when I notice diarrhea among my children and other members of my household is to visit the drug store to buy ORS. Because the ORS is usually the prescription given by the health centers when we visit, I do not waste my time going there. It is also cheaper to buy from a drug store than going to the hospital.”*

Another discussant drew the research team’s attention to home-remedies’ importance as a first management action for diarrhea.

*“I do not waste my time or money going to the health center or drug store to manage diarrhea. I know several home-made remedies, including mixing salt and ash, using ripe pawpaw (Carica papaya) and seeds. Even the neem leaves and barks (Azadirachta indica) can be an effective remedy if done right. I learned these from my grandmother.”*

The survey team asked a follow-up question on the type of approved pharmaceutical drugs used to manage diarrhea. The results suggest that most households (84%) in the studied communities use ORS to manage diarrhea, with the remaining 15.2% reporting Zinc supplements as the approved drug (Figure 4).

Among the communities, Anyanui-Atiteti has the highest ORS usage, with 90% of households using it. Anyanui-Atiteti is closely followed by Anyako, with 89.0% usage. Opetekwei (25%) and Mumford (15%) have the highest Zinc supplement usage. One woman discussant in the FGD emphasized the relevance of ORS for managing diarrhea.

*“I have known for several years that ORS is the main medicine for managing diarrhea. Therefore, I go straight to the drugstore that is common in the community to buy it. It is also not expensive. Sometimes, I get the Zinc when ORS is not available because I have been told by the nurse that it prevents diarrhea.”*

#### 3.3.2 Factors influencing diarrhea management behavior

Table 5 illustrates the various factors influencing the choice between modern pharmaceutical drugs and traditional herbal remedies for managing diarrheal diseases in different communities. The results highlight the significance of trust, affordability, availability, and religious and cultural factors in shaping individuals’ preferences for these treatment options. Regarding the use of modern pharmaceutical drugs in the Anyako, Anyanui-Atiteti, Opetekwei, and Mumford communities, trust significantly influences the use of modern practices for managing diarrhea (*p* < 0.001). In the Anyako and Anyanui-Atiteti communities, affordability is highly significant in influencing the use of modern pharmaceutical drugs (*p* < 0.001). In Opetekwei, it remains statistically significant (*p* < 0.01). However, in Mumford, it is not statistically significant (*p* ≥ 0.05). In the Anyako community, availability significantly influences modern pharmaceutical drug usage (*p* < 0.05). However, in the other communities, it is not statistically significant (*p* ≥ 0.05). In the Anyako community, religious and cultural factors significantly influence modern pharmaceutical drug usage (*p* < 0.05). However, in the other communities, they are not statistically significant (*p* ≥ 0.05). The results from the men’s focus group discussions in Anyako and Anyanui-Atiteti provided further evidence supporting affordability as a significant factor affecting the use of pharmaceutical drugs. In the words of a 60-year-old discussant.

**Table 5:** Factors influencing households’ use and dependence on approved pharmaceutical and traditional herbal remedies for diarrhea management in the studied communities.

Community	Factors influencing approved pharmaceutical (modern) drugs	*p*-value	Factors influencing traditional herbal remedies	*p*-value
Anyako	Trust	0.0001***	Trust	0.547
	Affordability	0.0012***	Affordability	0.002**
	Availability	0.019*	Availability	0.027*
	Religious and culture	0.01*	Religious and culture	0.001*
Anyanui-Atiteti	Trust	0.0001***	Trust	0.547
	Affordability	0.0***	Affordability	0.0075***
	Availability	0.379	Availability	0.027*
	Religious and culture	0.0***	Religious and culture	0.025*
Opetekwei	Trust	0.0001***	Trust	0.547
	Affordability	0.0***	Affordability	0.002**
	Availability	0.333	Availability	0.027*
	Religious and culture	0.033*	Religious and culture	0.045*
Mumford	Trust	0.0001***	Trust	0.547
	Affordability	0.131	Affordability	0.002**
	Availability	0.199	Availability	0.027*
	Religious and culture	0.169	Religious and culture	0.045*

*p* < 0.05: *(statistically significant, 95% confidence); *p* < 0.01: **(highly statistically significant, 99% confidence); *p* < 0.001: ***(very highly statistically significant, 99.9% confidence). Source: Field Survey, 2022.

*“As for my household, we do not have the money to go and buy drugs from the store. This is because drugs can be very expensive and sometimes do not even work effectively. I would prefer to save my little money to buy food for my family. I must also state that the local herbs can be effective for managing diarrhea. I prefer that.”*

The impact of these factors on the utilization of traditional herbal remedies varies across different communities (Table 5). Trust does not significantly influence traditional herbal remedies in all communities (*p* ≥ 0.05). However, affordability plays a highly significant role in the Anyako, Anyanui-Atiteti, and Mumford communities (*p* < 0.01).

Similarly, in Opetekwei, affordability remains statistically significant (*p* < 0.05). Availability also demonstrates a significant influence in the Anyako, Anyanui-Atiteti, and Mumford communities (*p* < 0.05), while in Opetekwei, it is statistically significant (*p* < 0.05). Religious and cultural factors have a significant impact on the use of traditional herbal remedies in the Anyako community (*p* < 0.05). Still, they are not statistically significant in the other communities (*p* ≥ 0.05).

## 4 Discussion

### 4.1 Socio-demographic characteristics, knowledge of causes and experiences of diarrhea

The results obtained from this study offer valuable insights into the socio-demographic and environmental factors that may influence the occurrence, knowledge, and households’ experiences of diarrheal diseases in the communities surveyed. The study findings indicate significant differences in demographic characteristics among households and communities in coastal Ghana (Table 2). The observed socio-demographic differences between households may be attributed to known variations in lifestyle, values, and social norms, particularly when comparing rural communities (Anyanui-Atiteti and Anyako) with urban settings (Mumford and Opetekwei). As other studies (52, 53) have found out, it is evident that urban areas, when compared to rural ones, have improved access to education, higher income levels, and a more robust provision of essential services, including clean water, sanitation facilities, and healthcare. On the other hand, rural households are typically more rooted in traditional, social, and cultural practices. The findings that males head a greater proportion of surveyed households in all coastal communities reflect the patriarchal nature of most communities and households in Ghana (54, 55). Men leading decision-making and having greater control over economic resources than women could significantly impact the prevention, control, and management of diarrheal diseases in coastal communities (56, 57).

The findings indicate that households with higher levels of formal education better understood proper hygiene and sanitation practices to prevent diarrhea. However, the study revealed a lower proportion of households that had completed tertiary education, indicating a potential need for increased educational opportunities to improve diarrhea health literacy in these communities. Furthermore, cultural and religious factors significantly influence the use of herbal medicine for managing diarrhea. Specifically, the Anyako community, with a higher proportion of traditionalists, demonstrated a greater utilization of herbal medicine for diarrhea management (Figure 4; Table 5). This suggests a strong relationship between traditional beliefs and the adoption of herbal and related practices for tackling diarrhea. These findings align with other studies (58, 59), highlighting the significant role of cultural beliefs and practices, as well as the influence of traditional healers, in shaping community responses to diseases including diarrhea.

Access to safe drinking water is crucial in preventing diarrheal diseases, which pose significant public health challenges in developing countries, including Ghana. The findings indicate the use of sachet water as the primary drinking water source (Table 3). Sachet water, often produced and packaged under poor hygienic conditions, poses a higher likelihood of contamination, contributing to diarrhea incidence (60). Conversely, the use of bottled water was relatively low, possibly due to the higher cost, which limited access to safe drinking water. In contrast, the use of pipe-borne water for drinking, cooking, and handwashing was associated with a lower diarrhea incidence, highlighting the importance of access to safe water sources in preventing diarrheal diseases (61–63). The risk of contamination in sachet water, as documented in earlier studies (64, 65), emphasizes the need for improved production and packaging standards to ensure the safety of this commonly used water source in coastal communities.

**Figure 5:**
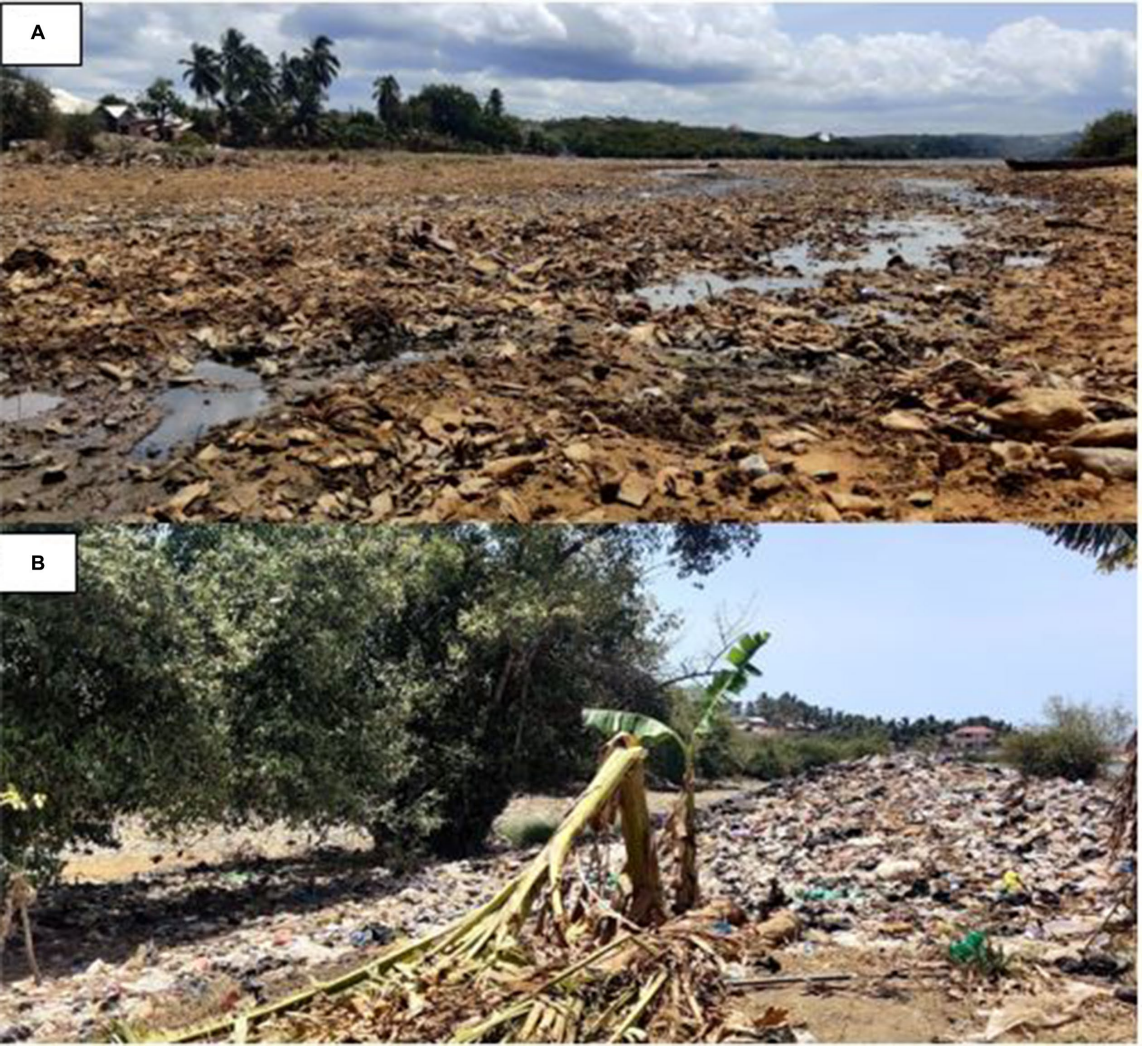
Accumulated waste polluting the lagoon ecosystem in one of the studied communities **(A)** waste accumulation destroying the lagoon ecosystem **(B)** Portion of the lagoon ecosystem used as a dumping ground.

From the study findings, the use of refuse dumps (open spaces) as the main solid waste management method in the studied communities could increase the risk of diarrhea as it provides a breeding ground for disease-causing bacteria and other pathogens. Our observation during the field survey revealed a concerning practice of open dumping of refuse, with waste materials polluting water sources, soil, wetland, and mangrove ecosystems across the studied communities. In Mumford and Anyako, for example, we observed poor sanitation and hygiene conditions due to excessive waste dumping in open spaces, including drainage systems (Figures 5A,B). Daily, we observed random dumping of domestic waste into nearby aquatic ecosystems, including lagoons, salt marshes, mangroves, and estuaries. In addition, open burning of this waste, which was mostly observed in Anyanui-Atiteti, can also release harmful pollutants into the air, which can cause or exacerbate respiratory problems, including coughing and asthma. Using refuse dumps as a waste management method has been associated with an increased risk of diarrhea (66, 67). These dumpsites often become breeding grounds for harmful pathogens and pests. When these pathogens come into contact with sources of drinking water or food, they result in contamination.

The results from the multivariate logistic regression analysis shows the complex interplay of factors influencing health outcomes in the studied communities. As the study has shown, respondents who disagreed with drinking from the river had significantly lower odds of experiencing diarrhea, consistent with studies emphasizing the critical role of safe drinking water in preventing diarrheal diseases (68–70). Conversely, disagreement about covering food was associated with higher odds of diarrhea, showing the importance of food hygiene practices in reducing foodborne illnesses. Similarly, those who disagreed that food was stored well had lower odds of diarrhea, reinforcing the need for proper food storage practices. Additionally, disagreement about the effects of flooding significantly increased the odds of diarrhea, highlighting the impact of environmental factors on health (71–73). The marked increase in odds of diarrhea among those with no idea about eating unwashed fruits suggests significant knowledge gaps regarding food safety.

The results from the second model, which revealed that males who disagreed with drinking from the river had significantly lower odds of experiencing diarrhea, highlighting gender differences in water use practices (74). Both males and females who disagreed about covering food had higher odds of diarrhea, indicating the universal importance of food hygiene. Females who disagreed with proper food storage had significantly lower odds of diarrhea, suggesting targeted interventions focusing on women’s roles in food handling could be effective. Disagreement about the effects of flooding significantly increased the odds of diarrhea for both males and females, emphasizing the need for improved flood management and sanitation infrastructure (75–78). The findings examining households with no formal education, showed higher odds of diarrhea among those who disagreed with drinking from the river. This points out the significant role education plays in determining and understanding health behaviors (79–81).

The above findings highlight the need for improving water quality and access, promoting food hygiene practices, and enhancing flood management and sanitation infrastructure to reduce the incidence of diarrheal diseases in coastal communities. Targeted educational interventions, particularly those addressing gender differences and the needs of individuals with lower education levels, are essential. Addressing critical knowledge gaps in food safety through community-based education programs can further mitigate the risk of foodborne illnesses.

### 4.2 Diarrhea management practices in study communities

From the study results, the high utilization of ‘over-the-counter’ (OTC) drugs as the first management action against diarrhea (Figure 3) aligns with previous research (21, 82) and is not surprising considering their ready availability in pharmacies, licensed chemical shops or informal markets. Economic constraints emerged as a critical factor influencing the choice between modern pharmaceuticals and traditional remedies. Participants from all communities highlighted affordability as a key consideration, with many expressing a preference for traditional remedies due to the perceived high cost of modern medicines. A participant from Mumford pointed out, ‘Sometimes, it’s just cheaper and more convenient to use what we have at home than to go to the pharmacy’. Some plausible reasons for preference for over-the-counter drugs may include a sense of self-reliance and familiarity and limited healthcare options. In urban studied communities like Mumford and Opetekwei, which reported the highest OTC use, the abundance and high concentration of drug stores and retail outlets, convenience, and cost-saving may be major drivers of households’ decision-making. Several studies have documented the prevalence of over-the-counter drug use as the primary management action for diarrhea. For example, a study by Saha et al. (83) in Bangladesh found that over-the-counter drugs among the study participants were the most common choice for diarrhea management. Similarly, a survey by Rangari et al. (84) in Andhra Pradesh, India reported that over-the-counter medications were widely used for self-treatment of diarrhea.

The finding that a low percentage of households initially seek care at health facilities for diarrhea management aligns with the literature emphasizing the roles of accessibility, cost, and perceptions of the adequacy of over-the-counter medications in influencing healthcare-seeking behaviors (85, 86). Notably, the enhanced propensity of households in Opetekwei and Anyako to utilize health facilities can be attributed to the availability of accessible healthcare infrastructure, such as Anyako Health Center, and the proximity to urban centers offering a variety of healthcare services. This behavior reflects broader public health insights that geographical accessibility and the presence of healthcare options significantly encourage the utilization of professional medical services (87). Such trends are crucial for improving diarrhea management outcomes, as seeking professional healthcare ensures accurate diagnosis and appropriate treatment, reducing the risk of complications and disease transmission (88). Therefore, enhancing healthcare infrastructure and accessibility, along with reducing barriers and increasing awareness about the importance of professional medical consultation, are vital steps toward promoting better health-seeking behaviors and outcomes in communities, especially for managing conditions like diarrhea.

The widespread reliance on home-made traditional (herbal) remedies for managing diarrhea in communities like Anyanui-Atiteti and Anyako underscores the deep-rooted tradition and preference for natural treatment methods. This practice is not unique to these communities; similar findings were observed by Yeung et al. (89) in Hong Kong, where traditional Chinese herbal medicine remains a common household remedy for diarrhea. Our survey corroborates these findings, indicating a widespread preference for herbal remedies attributed to their natural properties and ease of access, presenting them as viable alternatives to approved pharmaceutical drugs. This preference is further supported by literature, including studies by Adongo et al. (90) and Eshete and Lulekal Molla (91), as well as earlier research by Matu and van Staden (92), all of which highlight the continued popularity and perceived efficacy of these traditional treatments. An interaction with a key informant in Anyanui-Atiteti sums up the widespread use of home-made traditional remedies.

*“As an herbalist in Anyanui-Atiteti, I have witnessed the remarkable effectiveness of herbal medicine in treating diarrhea. Our traditional remedies, derived from natural plants and herbs abundant in our local environment, have been passed down through generations. These remedies harness the healing properties of nature to alleviate symptoms and restore balance to the body. Diarrhea is a common disease, so one does not need to go to the hospital. I firmly believe that embracing our rich herbal heritage can provide a safe and effective alternative for those seeking relief from diarrhea while also honoring our ancestral wisdom and connection to nature.”*

Our findings revealed valuable insights into the utilization of approved pharmaceutical drugs for managing diarrhea in the studied communities. It is encouraging to observe that most households employ oral rehydration salts (ORS) as the recommended treatment for diarrhea (Figure 4). ORS is widely recognized for its effectiveness in preventing dehydration, a common complication of diarrhea (14, 93, 94). The high usage rates observed in communities such as Anyanui-Atiteti and Anyako indicate a strong acceptance and adherence to this evidence-based practice. On the other hand, the relatively higher usage rates of Zinc supplements in communities like Opetekwei and Mumford suggest an awareness of its benefits and availability. Based on this finding, the study proposes a nationwide policy in Ghana to expand and enforce ORS distribution programs, mandating the availability of ORS in healthcare facilities. This policy should be accompanied by public education campaigns aimed at reducing unnecessary antibiotic use for diarrhea treatment.

### 4.3 Factors influencing diarrhea management behavior

Our results indicate that trust is the most reported factor influencing the use of approved pharmaceutical (modern) drugs (ORS and zinc supplements) and traditional herbal remedies for the management of diarrhea across the studied communities (95–98) (Table 5). In the Anyako community, trust was found to be a crucial factor influencing the use of pharmaceutical drugs, indicating that individuals in this community have confidence in the effectiveness and safety of modern medical treatments. However, trust did not significantly influence the use of traditional herbal remedies, suggesting that cultural and traditional beliefs may play a more prominent role in decision-making regarding herbal remedies (99). Affordability significantly influenced pharmaceutical drugs and traditional herbal remedies in the Anyako community. This suggests that treatment costs play a crucial role in decision-making, as individuals consider the financial implications of choosing between modern medicine and herbal remedies. Availability was also found to influence the choice of treatment in Anyako, with both pharmaceutical drugs and traditional herbal remedies being impacted by the accessibility of these options. Based on this finding, it is vital to ensure the consistent availability of both medicines to households within the Anyako community year-round.

In the Anyanui-Atiteti community, trust and affordability were found to be significantly influencing both approved pharmaceutical (modern) drugs and traditional herbal remedies. Availability did not significantly influence treatment choices for approved pharmaceutical drugs, but it remained significant for traditional herbal remedies. This finding emphasizes the importance of trust in management practices for diarrhea in Anyanui-Atiteti and the consideration of costs when selecting treatment options. It is also worth noting that, like Anyako, religious and cultural factors were highly significant for approved pharmaceutical drugs and traditional herbal remedies (Table 4).

For the Opetekwei and Mumford communities, trust and affordability were again found to influence pharmaceutical drugs and traditional herbal remedies significantly. This indicates that trust in healthcare providers and the affordability of treatment options are critical factors in these communities. In Opetekwei and Mumford, religious and cultural factors were moderately significant for approved pharmaceutical drugs and traditional herbal remedies. This suggests that cultural beliefs and practices play an important role in shaping the decision-making process for households seeking alternative healthcare options despite the availability and reliance on modern drugs (100, 101).

Through thematic analysis of the FGD transcripts, several key themes emerged, which include trust in traditional medicine, the impact of socio-economic factors on health-seeking behavior, and the influence of cultural and religious beliefs on the choice of diarrhea management practices. Participants from both rural and urban settings expressed a deep trust in traditional remedies for managing diarrhea, citing their efficacy, accessibility, and alignment with cultural beliefs. This preference was particularly pronounced in Anyako and Anyanui-Atiteti, where traditional medicine is deeply embedded in the community’s way of life.

The study’s findings on the factors affecting diarrhea management practices in coastal Ghana add to what is already known about the stark differences between urban (Mumford and Opetekwei) and rural (Anyako and Anyanui-Atiteti) communities. These differences include social structures, demography, infrastructure and services, and access to basic needs, among other things. From our study findings, it has become clear that Anyako and Anyanui-Atiteti households may have a stronger adherence to traditional beliefs and practices, a higher level of trust in traditional systems of healing, and an appreciation of traditional practices as integral to preserving cultural identity and heritage compared to urban households in Mumford and Opetekwei, which are more urban areas. Other studies have reported that rural households and communities prefer traditional herbal remedies for managing diarrhea (101–103).

## 5 Conclusion

This study contributes to understanding diarrhea management within climate change-vulnerable coastal communities in Ghana. The findings emphasize the significance of socio-demographic and environmental factors in shaping the incidence of diarrheal diseases. The study highlights the influence of education on understanding proper hygiene and sanitation practices, indicating the need for increased educational opportunities to improve diarrhea health literacy. The dominant use of sachet water as the primary drinking water source across the studied communities from previous studies is associated with a higher risk of diarrhea due to potential contamination. These findings emphasize the importance of ensuring access to safe water sources to prevent diarrheal diseases. Additionally, practices of open dumping of waste were observed in the studied communities. Open dumping provides a breeding ground for bacteria and pathogens, contributing to the spread of diarrheal diseases. Improving waste management practices and promoting proper disposal methods is important to mitigate the health risks associated with indiscriminate waste dumping. The study findings indicate that improving water quality, promoting food hygiene, and enhancing flood management and sanitation infrastructure are crucial for reducing diarrhea incidence.

The study findings indicate that over-the-counter drugs are widely used as the first response for managing diarrhea, particularly in urban communities. The convenience, accessibility, and cost-saving aspects of these drugs were reported as significant factors contributing to their popularity. Conversely, many households reported visiting healthcare facilities as their first management action, highlighting the importance of seeking professional healthcare services for appropriate diagnosis and treatment. Additionally, the study revealed the enduring popularity of traditional herbal remedies for diarrhea management, particularly in specific communities. The use of homemade traditional remedies reflects a reliance on perceived natural properties and accessibility as an alternative to pharmaceutical medications. The findings suggest a need for promoting appropriate healthcare-seeking behaviors to ensure timely and accurate diagnosis and treatment of diarrhea. Public awareness campaigns can educate communities about the importance of seeking healthcare services for effective management.

This study also highlights the importance of integrating traditional healing practices for diarrhea management in climate-vulnerable coastal communities in Ghana. Through recognition and regard for cultural beliefs, interventions can be formulated to facilitate the sustainable utilization of medical plants and herbs while cultivating community involvement and creating social cohesion. It is also important to emphasize that drawing on traditional medicine for diarrhea management aligns with the principles of nature-based solutions by utilizing natural resources, embracing holistic approaches, preserving cultural knowledge, and promoting accessibility and affordability.

As this study has found, to address the challenges of diarrhea management in coastal Ghana, a multi-sectoral approach is needed to address the disease’s underlying causes. This approach should include improved access to safe water and sanitation facilities, education on the causes and prevention of diarrhea, and adequate healthcare services within communities at all times. It is also essential to invest in training healthcare workers in appropriately managing diarrhea, including using ORS and zinc supplementation. Actively promoting good hygiene practices in community settings such as homes, schools, and public spaces, including lorry parks, churches, funeral grounds, and recreational areas, can reduce the incidence of diarrheal disease. Moreover, the enduring popularity of traditional herbal remedies underscores the importance of acknowledging and exploring the potential for integrating traditional knowledge with modern healthcare practices. Such integration may provide a culturally sensitive and comprehensive approach to managing diarrhea, ultimately improving these communities’ health outcomes.

## Data availability statement

The original contributions presented in the study are included in the article/supplementary material, further inquiries can be directed to the corresponding author.

## Ethics statement

The studies involving humans were approved by Ethics Committee for the College of Basic and Applied Science (ECBAS 044/19–20) of the University of Ghana and the Ghana Ministry of Health (GHS-ERC-011/11/20). The studies were conducted in accordance with the local legislation and institutional requirements. The participants provided their written informed consent to participate in this study.

## Author contributions

YB: Conceptualization, Data curation, Formal analysis, Funding acquisition, Investigation, Methodology, Project administration, Resources, Software, Supervision, Validation, Visualization, Writing – original draft, Writing – review & editing. FO: Conceptualization, Data curation, Funding acquisition, Investigation, Methodology, Project administration, Resources, Writing – review & editing. JA: Conceptualization, Data curation, Funding acquisition, Investigation, Methodology, Project administration, Resources, Supervision, Validation, Writing – review & editing. JAA: Data curation, Investigation, Methodology, Project administration, Resources, Supervision, Writing – review & editing. DY-T: Conceptualization, Data curation, Formal analysis, Funding acquisition, Investigation, Methodology, Project administration, Resources, Supervision, Validation, Writing – review & editing. AM: Conceptualization, Data curation, Funding acquisition, Investigation, Methodology, Project administration, Resources, Supervision, Writing – review & editing. CD: Resources, Supervision, Writing – review & editing, Conceptualization, Data curation, Funding acquisition, Investigation, Methodology, Project administration. TA: Writing – review & editing, Conceptualization, Funding acquisition, Investigation, Methodology, Project administration, Resources, Supervision. LA: Writing – review & editing.
